# Recasting Nitrogenase’s Carbide Role as a Beating Heart of Steel: A Joint Inorganic and Organic Perspective for μ_6_Carbide–Iron Bonding

**DOI:** 10.1021/acs.inorgchem.5c05356

**Published:** 2026-02-18

**Authors:** Justin P. Joyce, Serena DeBeer

**Affiliations:** † Department of Inorganic Spectroscopy, 28313 Max Planck Institute for Chemical Energy Conversion, Stiftstr. 34-36, Mülheim an der Ruhr D-45470, Germany

## Abstract

Nitrogenase’s cofactor features a trigonal prismatic interstitial carbide, an architectural motif without parallel in other biological systems whose enzymatic significance remains unclear. A ^13^C ENDOR study indicated negligible hyperfine coupling at the carbide, hinting at an inert geometric and electronic structure that preserves its trigonal prismatic framework and the antiferromagnetic coupling of the Fe-sites. Contrary to the "heart of steel" interpretation, the "beating heart" model proposes that structural flexibility aids cofactor stabilization during catalysis. Here, we establish the theoretical foundation of the carbide’s bonding using valence bond (VB) and molecular orbital (MO) theory, both indicating a preference for the trigonal prismatic geometry with antiferromagnetic coupling. The carbide in FeMoco’s resting state shows six equivalent σ-bonds with half bond order from sp^2^-hybridization, linking insights from inorganic and organic chemistry. Our findings, supported by broken-symmetry density functional theory (BS-DFT) and quantum mechanics/molecular mechanics (QM/MM) modeling, show that the carbide retains its trigonal prismatic geometry, while its local σ/π bonding and hybridization adapt to the spin coupling of the Fe-centers. Altogether, our findings suggest that the carbide imparts an inert geometric framework alongside a dynamic electronic structure that enables the catalytic reduction of diverse substrates.

## Graphical abstract

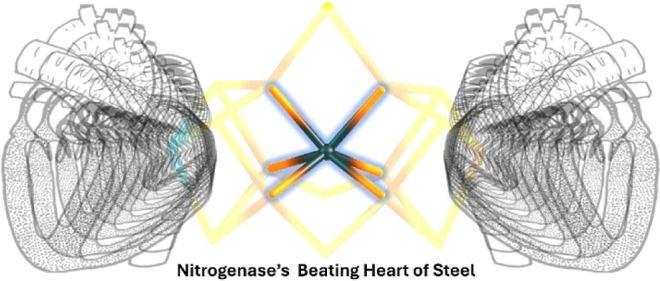

## Introduction

Mo-dependent, V-dependent, and Fe-only nitrogenases are enzymes named for their reduction of dinitrogen to ammonia, a biological analogue of the Haber–Bosch process.^1,2^ The enzyme’s substrate binding and activation site is a complex metallocofactor (FeMco) composed of fused Fe–S hetero- and homocubane clusters bridged by an interstitial, trigonal prismatic carbide (Figure 1).^3^ These FeMco clusters are distinguished by an octahedral, homocitrate-bound metal site (M = Mo, V, Fe) and by a μ_2_-ligand (X = sulfide or carbonate) that bridges the hetero- and homocubane subclusters. As established by X-ray spectroscopy, the unifying structural feature of nitrogenase is the central carbon that bonds to six Fe-groups.^4–6^ The mutation of amino acid residues belonging to the secondary coordination sphere of FeMoco’s CFe_6_ core suggests that it is the site for nitrogen fixation.^7,8^ Similarly, crystallographic studies in all nitrogenase cofactors have shown the substitution of the clusters’ μ_2_-sulfides, suggesting its lability promotes N_2_ binding.^9–16^ While hexavalent carbon has not been observed in biological systems outside of nitrogenase, its catalytic significance is unknown.

**Figure 1:**
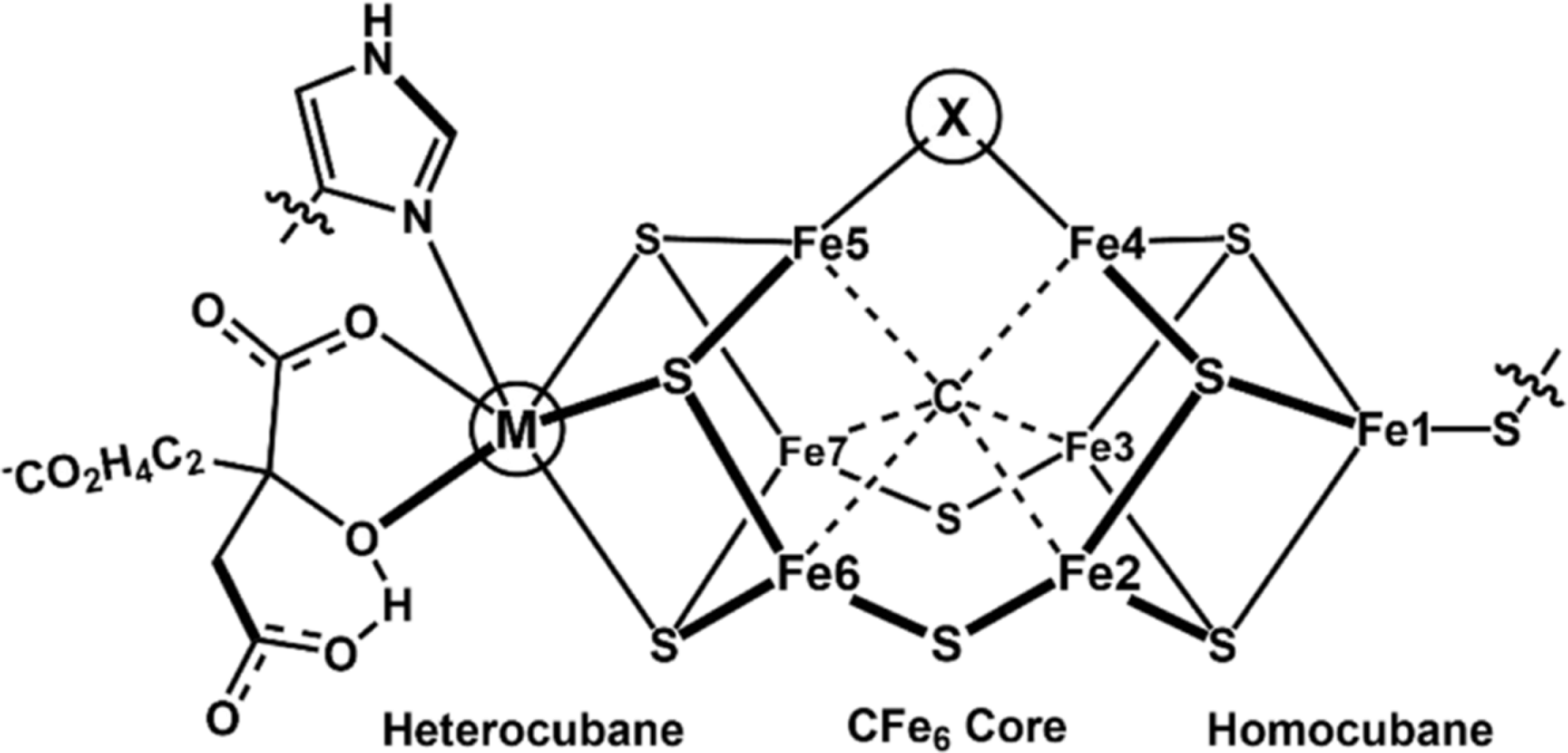
Structure for the nitrogenase clusters that includes FeMoco (M = Mo^3+^, X = S^2–^), FeVco (M = V^3+^, X = CO_3_^2–^), and FeFeco (M = Fe^2+/3+^, X = S^2–^).

Coined by Einsle^17^ and popularized by Hoffman,^18^ two competing narratives have emerged that portray the central carbon as either the “beating heart” or “heart of steel” of nitrogenase, representing, respectively, a dynamic core or inert structural framework that facilitates the dramatic transformation of nitrogen to ammonia. Supporting the “beating heart” interpretation, Peters proposed, based on his molecular model complexes, that the carbide in nitrogenase facilitates N_2_ binding through fluxional coordination modes.^19,20^ With respect to the enzyme, Nuclear Resonance Vibrational Spectroscopy (NRVS) shows changes in the symmetric μ_6_-carbide “breathing mode” of FeVco when compared to FeMoco and FeFeco, which we attributed to FeVco’s distinct carbonate bridging ligand.^21^ Cramer similarly identified a change in this NRVS feature for FeMoco’s CO-bound state, which was attributed to a terminal coordination mode *trans*- to the central carbide, as later supported by crystallographic studies.^22–24^ Consistent with these observations, nitrogenase’s Fe–C force constant of 1.31 N cm^–1^, approximately half the value for representative Fe–CO–carbide clusters, further underscores the comparatively flexible bonding of the CFe_6_ core.^25,26^

Einsle’s favored “heart of steel” interpretation postulated that nitrogenase’s carbide enforces rigidity between the bridged Fe-centers, thereby destabilizing the μ_2_-bridging hydrides of the proposed E_4_ state, which in turn facilitates reductive elimination and N_2_ binding and activation.^17^ Einsle’s carbide categorization scheme has since been adopted by Hoffman, based on their ^13^C ENDOR spectra of the selectively labeled carbide in FeMoco’s E_0_, E_2_, and E_4_ states, as well as in its CO and alkyne-bound forms.^18,27^ The near-zero ^13^C hyperfine coupling observed in these spectra was interpreted as a net cancellation of the individual Fe–carbide bond spin dipoles, with their covalency effectively masked by the cofactor’s overall exchange-coupling network. We have since provided direct support for their interpretation by characterizing the ^13^C hyperfine coupling in an asymmetric μ_4_-carbide Fe–S cubane, which confirms the presence of strong Fe–carbide bonding.^28^

Currently, no molecular bonding framework directly connects the six-coordinate carbide in nitrogenase’s core to the familiar valency concept of organic chemistry. Although predating quantum mechanics, the tetravalency of carbon-based molecules, as predicted by Lewis’ octet rule, remains a fundamental principle of organic structure and reactivity.^29^ This principle is consistent with valence bond theory, which describes carbon’s electronic structure in terms of hybridization between its 2s and 2p valence electrons.^30^ The functional groups that comprise the organic chemistry toolbox are neatly classified by their hybridization: sp (alkynes and nitriles), sp^2^ (alkenes, arenes, and carbonyls), or sp^3^ (alkanes) as shown in Figure 2 (top). Instances in which carbon exceeds four bonds are rarely encountered outside the context of the five-coordinate transition state connecting the reactants and products of S_N_2 substitution reactions.^31^ Challenging this conventional view, Schleyer argued that carbon’s hypervalency should be considered as a general rule, citing well-characterized examples such as carbide carbonyl clusters, carboranes, and carbocations. He referred to these species as “embarrassing compounds”, orphaned in the organic field and “relegated to the inorganic world” that are shown in Figure 2 (bottom).^32^

**Figure 2:**
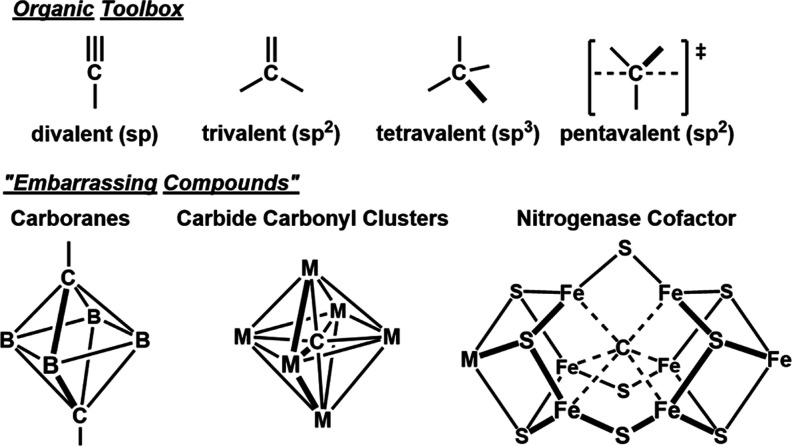
(Top) Examples of carbon valency in organic systems; (bottom) examples of carbon valency exceeding four for inorganic systems that are titled in reference to Schleyer’s description.^32^

Herein, we derive a molecular orbital (MO) and valence bond (VB) description for nitrogenase’s interstitial carbide. Ultimately, we find that the carbide provides a flexible electronic structure and rigid geometric structure that are consistent with both the “beating heart” and “heart of steel” schools of thought.

## Methods

Our QM/MM model for FeMoco is based on the 1.0 Å resolved crystal structure (PDB 3U7Q) that has been previously detailed.^33,34^ The QM/MM program ASH was used,^35^ providing a flexible interface to the OpenMM molecular mechanics library^36^ and the ORCA quantum chemistry code (ORCA v5.03).^37,38^ The covalent QM/MM boundary was treated with link atoms and charge-shifting.^39^ Geometries were optimized with the geomeTRIC optimization library^40^ using HDLC coordinates^41^ of an active region, defined as all residues within a 10 Å radius from the interstitial carbide (959 atoms). The QM-region is 168 atoms, including link atoms, which constitute FeMoco’s primary and secondary coordination sphere: Val70, Arg96, Gln191, His195, Cys275, Ser278, Gly356, Gly357, Arg359, Glu380, Phe381, His442, and HOH519.

The r^2^SCAN density functional^42^ was utilized in combination with the D4 dispersion correction,^43,44^ and the ZORA scalar relativistic Hamiltonian,^45,46^ a protocol that has been benchmarked for the geometric and electronic structure of spin-coupled iron–sulfur-based systems.^47^ The relativistically recontracted ZORA-def2-TZVP basis set was used for the Fe and S centers and the interstitial carbide of the clusters.^48,49^ The all-electron SARC-ZORA-TZVP basis set was used for Mo.^50^ All of the remaining atoms used the smaller ZORA-def2-SVP basis set. The Split-RI-J approximation in ORCA was used together with a decontracted auxiliary basis set (“SARC/J”).^51,52^

The spin populations of select BS-solutions were calculated by Hirshfeld population analysis.^53^ The metal center oxidation and spin states and their magnetic interactions are analyzed with Pipek–Mezey (PM) localized orbitals.^54^ The localized spin density (ρ_α,β_) was taken as the difference of the squared PM-localized orbitals of the α- and β-spin. The carbide’s agreement with a trigonal prismatic coordination environment was quantified by the Continuous Shape Measure (CShM) using the SHAPE v2.1 program.^55^

The cofactor’s E_0_ state was calculated with broken-symmetry-density functional theory (BS-DFT) within the QM/MM model. The BS-solutions of the [MoFe_7_S_9_C]^1–^ cluster were found by inverting the net spin on specific Fe-sites from α- to β-spin, converging from an *S* = 35/2 to *M*_s_ = 3/2 state. The Moheterometal is not specified in the flip-spin procedure because its spin density is freely oriented to optimize the covalency with the neighboring Fe-sites. Our notation specifies the cofactor’s labeled metal centers of β-spin, with respect to its crystallographic formalism, that is connected to Noodleman’s notation in Table 1.^34,56,57^

**Table 1:** Corresponding Broken-Symmetry Solutions, for a Distinct *M*_s_-Solution of Nitrogenase’s Cofactor, with respect to Noodleman’s and Our Notation Schemes^56^

Noodleman notation	Our notation
BS1	BS-567
BS2	BS-234
BS3	BS-123
	BS-124
	BS-134
BS4	BS-257
	BS-356
	BS-467
BS5	BS-256, BS-267
	BS-357, BS-367
	BS-456, BS-457
BS6	BS-156
	BS-157
	BS-167
BS7	BS-235
	BS-247
	BS-346
BS8	BS-245, BS-345
	BS-236, BS-246
	BS-237, BS-347
BS9	BS-126
	BS-137
	BS-145
BS10	BS-127, BS-136
	BS-135, BS-147
	BS-125, BS-146

## Results and Discussion

### Broken-Symmetry Description

The distribution of α- and β-spin metal centers defined by FeMoco’s BS-wave function reflects the cofactor’s underlying ferro- and antiferromagnetic interactions, which in turn determine the resulting oxidation and spin states. Approximating the cofactor’s *C*_3_-symmetry yields ten distinct BS-solutions, labeled according to the notation introduced by Noodleman, as shown in Figure 3.^56^ The descent from *C*_3_-symmetry, which accounts for the cofactor’s homocitrate ligand and surrounding protein environment, increases the total number of BS-solutions to thirty-five, our notation assigning the metal centers with net β-spin (Table 1).^34,57^ BS-solutions in the same Noodleman class have similar energies but can produce distinct cofactor geometries and electronic structures, potentially influencing reactivity. The Mo-heterometal readily adopts either a local high-spin (*S* = 3/2) or “non-Hund” (*S* = 1/2) configuration, with its net spin density enabling covalent-type metal–metal bonding to the three adjacent Fe-centers.^58^

**Figure 3:**
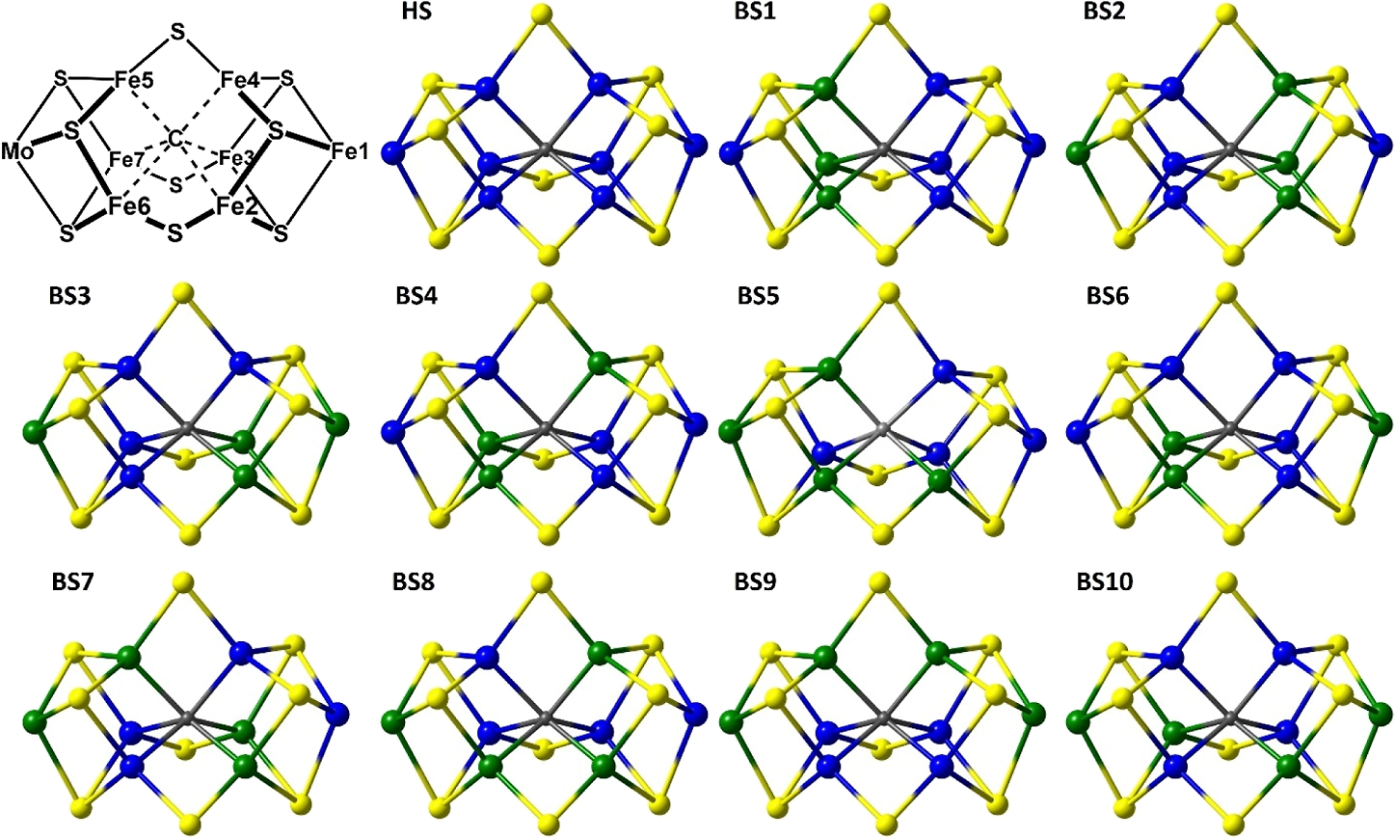
Ten BS-classes for the E_0_ resting state (*M*_s_ = 3/2) of FeMoco. The net α- and β-spin densities at the metal centers are blue and green, respectively. The homocitrate ligand and histidine residue coordinated to the Mo-heteroatom are omitted to show its *C*_3_-symmetry.

The relative energies of the Noodleman BS-classes for the E_0_ state of FeMoco are listed in Table 2, reported as the average of the corresponding BS-solutions arising from the cofactor’s descent from *C*_3_-symmetry that is included in Tables S1–S3. The average and standard deviation of the C–Fe bond lengths are also included and are 1.99 ± 0.01 Å, as determined from the 1.0 Å resolution crystal structure of FeMoco (PDB 3U7Q).^33^ We quantify the trigonal prismatic geometry of the carbide with the continuous shape measure (CShM), a unitless metric where a value of zero corresponds to an ideal geometry.^55^ We compare FeMoco’s crystallographic CShM value of 0.04 to that of the [Fe_6_(CO)_16_C]^2–^ cluster, whose value of 15.34 indicates a significant deviation from an ideal trigonal prismatic geometry, consistent with an octahedral coordination environment.^59^

**Table 2:** Relative Energies, Average Carbide–Fe Distances, and Trigonal Prismatic Continuous Shape Measure (CShM) for the QM/MM Optimized BS-Classes of FeMoco’s E_0_ State^a^

BS-Class	Energy (kcal mol^–1^)	$\overset{®}{d_{C-Fe}\;}$ (Å)	CShM (TPR-6)
BS1	30.4	1.98 ± 0.14	0.23
BS2	10.9	1.98 ± 0.07	0.18
BS3	25.0 ± 1.7	2.00 ± 0.08	0.21 ± 0.03
BS4	20.7 ± 11.4	1.99 ± 0.03	0.07 ± 0.05
BS5	22.7 ± 8.9	1.99 ± 0.06	0.13 ± 0.04
BS6	7.3 ± 0.3	1.99 ± 0.06	0.10 ± 0.01
BS7	0.0 ± 0.8	1.99 ± 0.01	0.03 ± 0.01
BS8	7.0 ± 1.0	1.99 ± 0.06	0.12 ± 0.01
BS9	12.2 ± 1.0	2.00 ± 0.06	0.08 ± 0.02
BS10	6.7 ± 0.8	2.00 ± 0.08	0.14 ± 0.03
PDB 3U7Q	-	1.99 ± 0.01	0.04
[Fe_6_(CO)_16_C]^2–^	-	1.88 ± 0.01	15.34

a The values of the standard deviations correspond to the distinct BS-solutions that belong to a BS-class included in Table 1. The structural parameters of FeMoco’s crystal structure (PDB 3U7Q) and a carbide carbonyl cluster, [Fe_6_(CO)_16_C]^2–^, are provided for reference.^33,59^

The BS-classes are partly distinguished by the magnetic coupling between Fe-sites in the hetero- and homocubane subclusters that share a common edge within the trigonal prism. Consistent with our previous reports, the BS7 class is the most stable, which can be attributed to the presence of three antiferromagnetic interactions between Fe-sites in the hetero- and homocubane subclusters that share a common μ_2_-bridging ligand.^60^ The BS8 and BS10 classes lie slightly higher in energy, at 7.0 ± 1.0 and 6.7 ± 1.1 kcal mol^–1^ above the BS7 ground state, respectively. Experimentally, the ^2,1^H ENDOR dipolar coupling tensors of FeMoco’s E_4_ state are consistent with either a BS8 or a BS10 solution, whereas the BS7 class is incompatible with the proposed dihydride structure.^61^ Our previous calculations, together with those of others, indicate that the BS10 class represents the energetic ground state for select E_4_ isomers and CO-bound states.^62–65^ Despite these energetic differences, the average C–Fe bond lengths and trigonal prismatic geometry are conserved across all BS-classes, which suggests the geometric structure is not directly indicative of the carbide’s electronic structure. Our manuscript focuses on the BS7 and BS10 classes, whose relative C–Fe bond lengths are illustrated in Figure 4.

**Figure 4:**
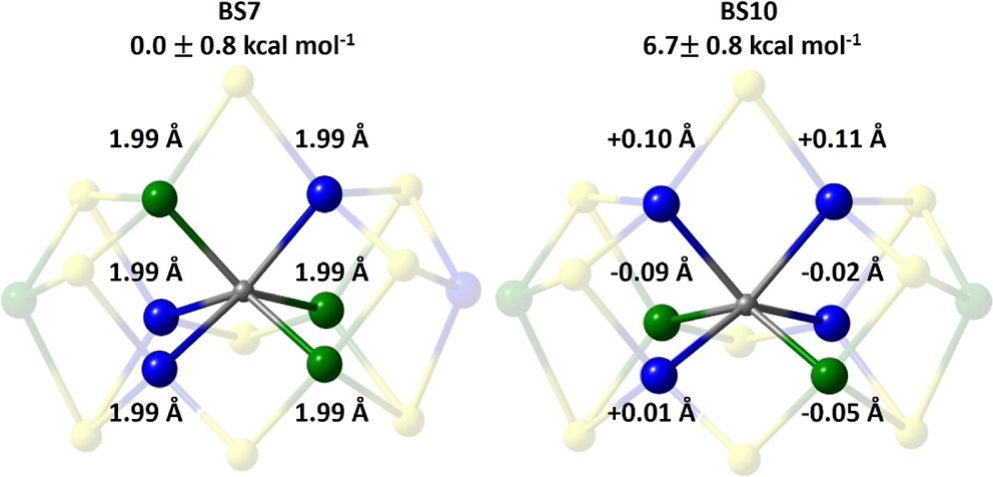
QM/MM optimized energies and C–Fe bond lengths for the BS7 (left) and BS10 (right) solutions for FeMoco’s E_0_ state. Both properties are reported as an average of the BS-solutions related by the cofactor’s *C*_3_-symmetry that is listed in Table 1. The Fe-sites with net α- and β-spin are shown as blue and green, respectively, highlighting the trigonal prismatic CFe_6_ core.

### Valence Bond Interpretation

The carbide has a fully occupied valence shell, incurring no energetic penalty for the hybridization of the s- and p-orbitals. The symmetry observed in carbide carbonyl clusters has been linked to the carbide’s hybridization in previous ^13^C NMR studies, with octahedral coordination in Fe-based examples being consistent with sp-hybridization.^66^ We have similarly assigned sp-hybridization to the μ_4_-carbide’s butterfly geometry in an asymmetric Mo–Fe–S cluster.^28^ The nitrogenase cofactor’s *D*_3h_ carbide core, as also observed in Rh carbide carbonyl clusters, is consistent with sp^2^-hybridization, as illustrated in Figure 5. Based on accompanying QM calculations, Hoffman assigned sp^2^-hybridization to nitrogenase’s interstitial carbide, proposing that it forms single-electron rather than electron pair covalent bonds with its coordinated Fe-centers.^18^ However, this model did not address how only three orbitals could be simultaneously directed toward the six Fe-sites of the core, nor did it account for the properties of the unhybridized p-orbital.

**Figure 5:**
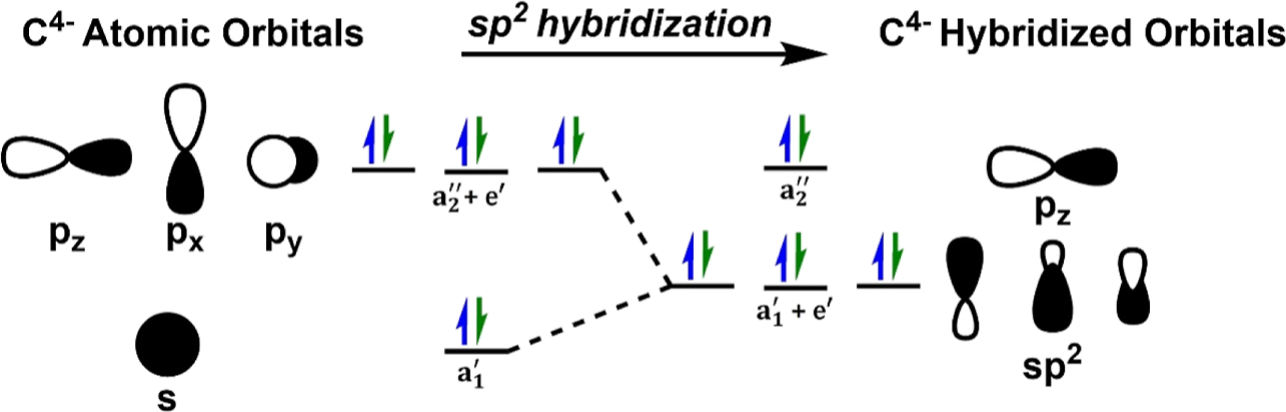
sp^2^-hybridization of the atomic carbide’s valence 2s and 2p orbitals with respect to FeMoco’s trigonal prismatic *D*_3h_ symmetric core.

The carbide’s hybridization is illustrated using Pipek–Mezey localized orbitals in Figure 6 for the lowest energy BS7 class. The carbide’s valence orbitals are fully occupied, exhibiting a_1_^′^(2s), e^′^(2p_*x*_ and 2p_*y*_), and a_2_^″^(2p_*z*_) symmetries with respect to the trigonal prismatic *D*_3h_ point group. The sp^2^-hybrids have a_1_^′^ and e^′^ symmetries, adopting a “propeller” arrangement within the cofactor core, with each orbital bisecting Fe-pairs along a trigonal prismatic edge. The remaining unhybridized p_*z*_ orbital is oriented along the cofactor’s *C*_3_ axis, lying within the fused cubane core. We note that similar magnetic axes were assigned to FeMoco’s E_0_ state by single-crystal EPR spectroscopy.^67^ Each carbide-centered orbital shows σ-bonds with the d_*z*_^2^ orbitals of the adjacent Fe-sites and minimal π-bonding, Figure 6 showing how the π-symmetric d_*xz*_ and d_*yz*_ orbitals overlap poorly with the carbide’s sp^2^-hybridized lobes.

**Figure 6:**
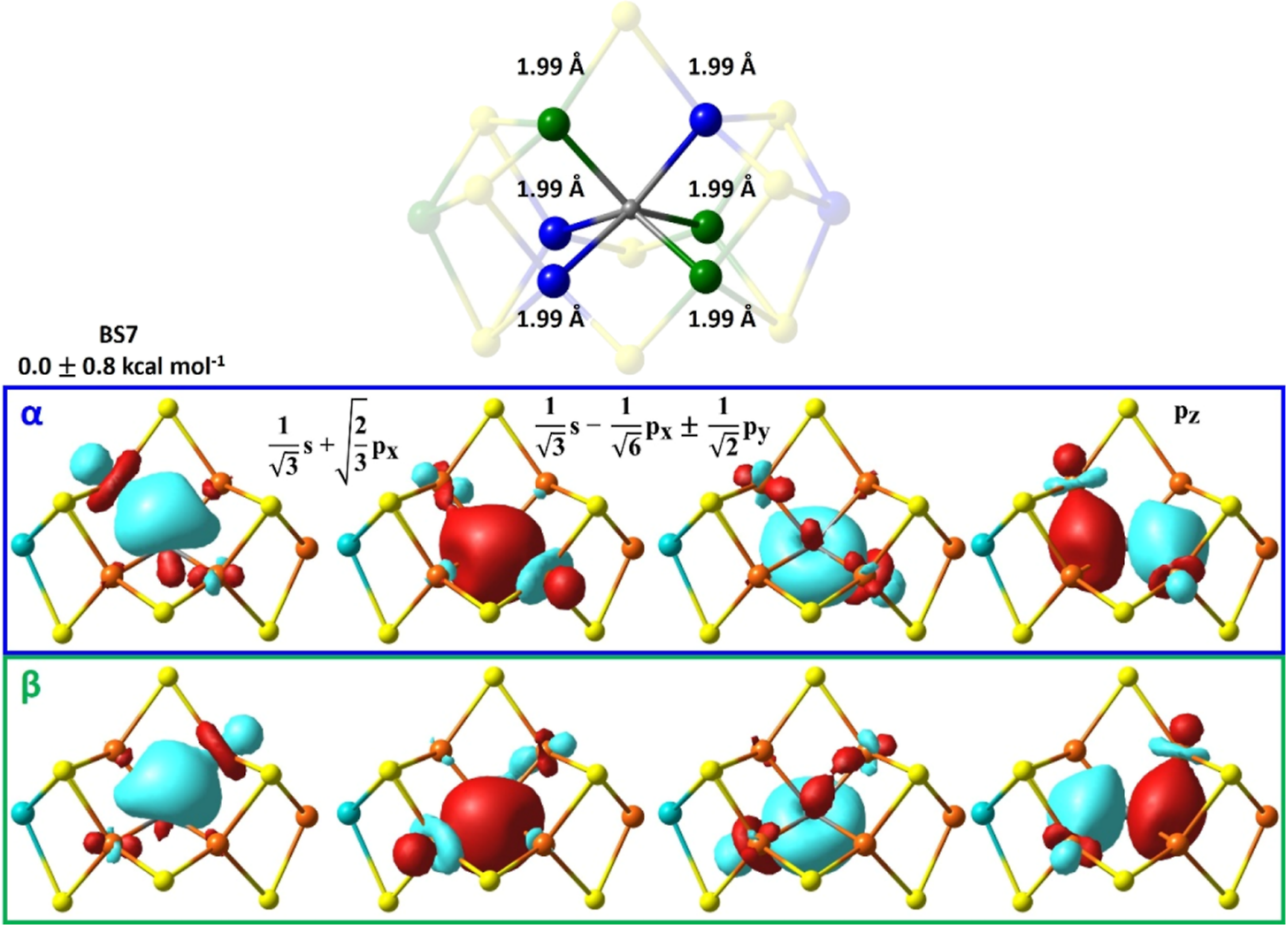
PM-localized orbitals of α-(top, blue) and β-(bottom*,* green) spin with Fe–carbide bonding character in FeMoco’s BS7 resting state, optimized with respect to the BS-235 solution. The linear combination of carbide’s atomic orbitals is provided for sp^2^-hybridization.

Figure 6 shows that the carbide-centered orbitals exhibit spin-polarization with α-spin electrons distorting toward coordinated Fe-sites of net β-spin and vice versa*.* We include the localized orbitals corresponding to one of the carbide’s lone pairs that bonds to the Fe-pairs (Figure 7). The four orbitals exhibit distinct α- and β-spin densities (ρ_α,β_), illustrating that the total spin-dipole of the carbide–Fe bonds is zero, consistent with Hoffman’s description. We include the molecular orbital (MO) diagram for this local bonding interaction, which is analogous to the three-center 4-electron (3c–4e) hypervalent bonding model and the Anderson superexchange model for antiferromagnetic coupling.^68^ We note that the spin specificity of the C–Fe bonds enables the carbide to form six bonds without violating carbon’s intrinsic valency, as described by Dunning’s recoupled bond pair dyad model for hypervalent molecules.^69,70^

**Figure 7:**
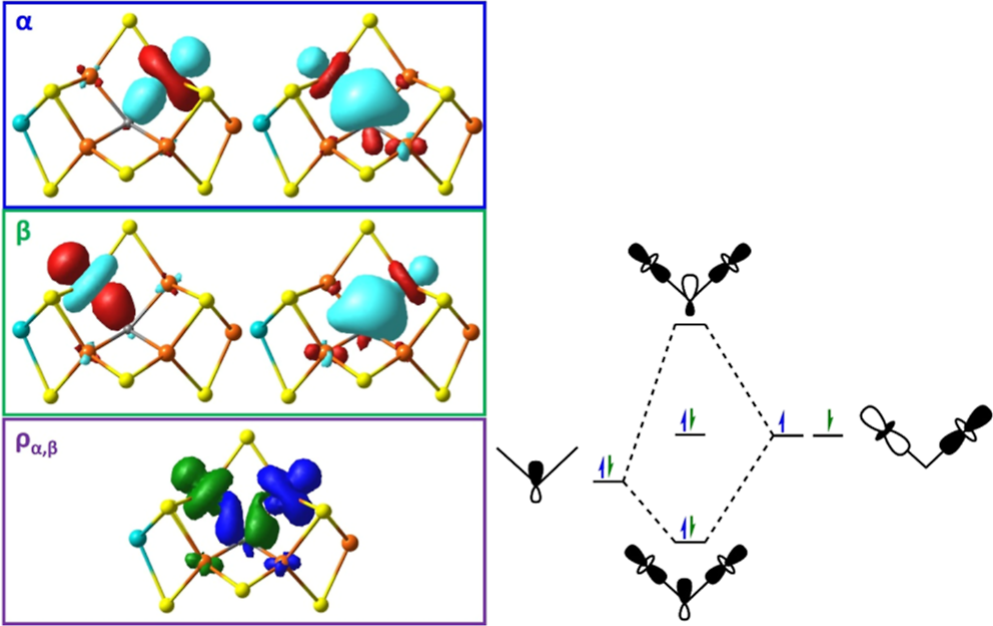
(Left) The localized orbitals of α- and β-spin for the carbide’s sp^2^ σ-bonding to an Fe-pair in FeMoco’s representative BS7 state (BS-235). The spin density (ρ_α,β_) of the four localized orbitals is provided, where α and β are, respectively, blue and green. (Right) The molecular orbital diagram from the three-center four-electron interaction of the carbide with the Fe-centers.

We emphasize that the six equivalent spin-polarized C–Fe σ-bonds require antiferromagnetic coupling between the Fe-sites bisected by the carbide’s sp^2^ orbitals. Comparatively, the BS10 solution has ferromagnetic coupling between a pair of Fe-sites that would suggest that it forms just four C–Fe σ-bonds. We include the BS10 solutions localized orbitals and their associated valence orbitals linear combinations in Figure 8 and Table S5 to illustrate how the μ_6_-carbide is adaptive to the magnetic coupling of the surrounding Fe-sites to compensate for the energetic loss of its spin-polarized σ-bonds. Since the carbide has no energetic penalty for rehybridization, one of its α-orbitals decreases its total p_*x*_ character to reduce overlap with the ferromagnetically coupled Fe-sites. The weakened σ-bonding between the carbide and the ferromagnetically coupled Fe-sites is reflected in the +0.11 Å elongation of their C–Fe bond lengths. Concurrently, the total p_*x*_-character of the remaining α-orbitals increases, enhancing overlap with the Fe-centers’ π_⊥_ symmetric d_*yz*_ orbitals and facilitating the formation of C–Fe π-bonds that provide an alternative source for stability. Because there are more α- than β-spin Fe-centers, the π-bonding is spin-specific with the α-electrons polarizing toward Fe-sites that possess net β-spin density. These π-bonding interactions are seen in the average −0.07 Å contraction of the C–Fe bond lengths associated with Fe-sites of net β-spin. The carbide’s orbitals with β-spin similarly exhibit the sp^2^-hybridization seen in the BS7 solutions, the linear combinations of the carbide’s atomic orbitals listed in Figure 8. We note that despite the distinct bonding regimes, the BS7 and BS10 solutions have μ_6_-carbide spin densities of 0.00 and 0.01α, respectively, that would be consistent with the carbide’s negligible hyperfine coupling.

**Figure 8:**
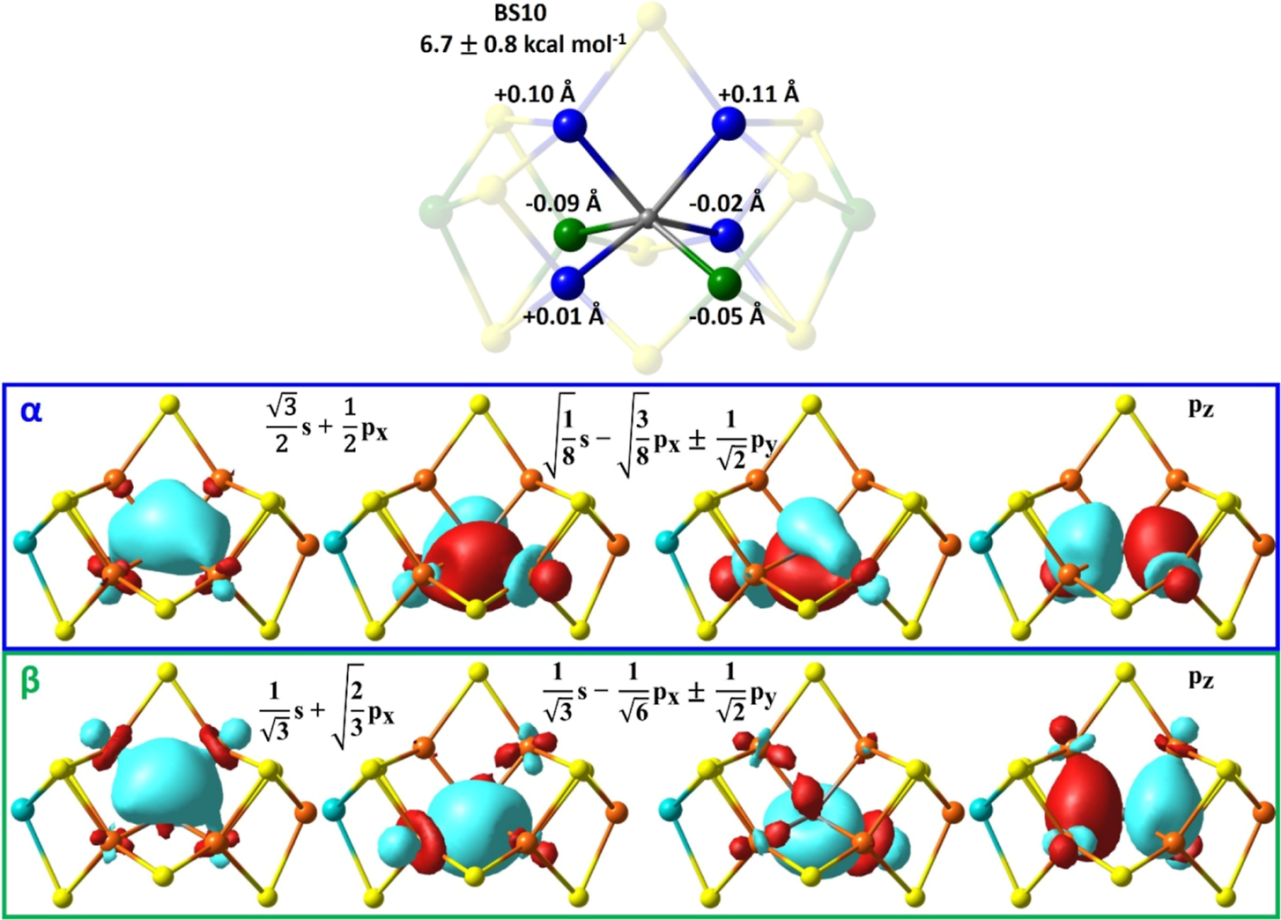
PM-localized orbitals of α-(top, blue) and β-(bottom, green) spin with Fe-carbide bonding character in FeMoco’s BS10 resting state. The linear combination of carbide’s atomic orbitals is provided and its population analysis in Table S4.

### Molecular Orbital Interpretation

The coordination environments of the interstitial Fe-sites are intermediate between tetrahedral and trigonal pyramidal geometries, with their local *C*_3_ axes oriented along the Fe–carbide bonds.^71^ The symmetry adapted linear combinations (SALCs) of the Fe-centers’ d_*z*_^2^ orbitals form σ-bonds with the carbide core, exhibiting a_1_^′^, e^′^, a_2_^″^, and e^″^ symmetries, as shown in Figure S2. The local oxidation states of the Fe-centers range from +2 to +3, with singly occupied d_*z*_^2^ orbitals in the high-spin configurations whose α- or β-spin is determined by the cofactor’s broken-symmetry wave function. The d_*yz*_ orbitals can form perpendicular π-bonds with the central carbide and exhibit a_2_^′^, e^′^, a_1_^″^, and e^″^ symmetries, with their total occupation determined by the ferromagnetic mixed-valence interactions between the Fe-centers.

This MO description of the CFe_6_ core (Figures 9 and 10) is consistent with the Fe valence-to-core X-ray emission spectra (VtC XES) that we have previously reported for all nitrogenases.^4–6,72^ The occupied a_1_^′^ bonding orbital, primarily of carbon 2s character, corresponds to the spectrum’s Kβ’’ transition, the diagnostic feature for a metal’s primary coordination environment. The Kβ_2,5_ feature of the VtC XES arises from ligand *n*p to metal 1s transitions and reflects, in part, the contributions of the carbide’s occupied e^′^ and a_2_^″^ bonding orbitals. Detailed above, the BS7 solutions primarily have C–Fe σ-bonds whose coordinated Fe-sites have equal net α- and β-spin densities. The core’s nonbonding e^″^ and antibonding a_1_^′^ orbitals are occupied along with the four bonding orbitals, resulting in an isotropic electronic state. The six resulting Fe–C bonds are equivalent, giving individual Fe–C bond orders of $\frac{1}{2}$ , which is consistent with our valence bond description of spin-polarized σ-bonds shown in Figure 7.

**Figure 9:**
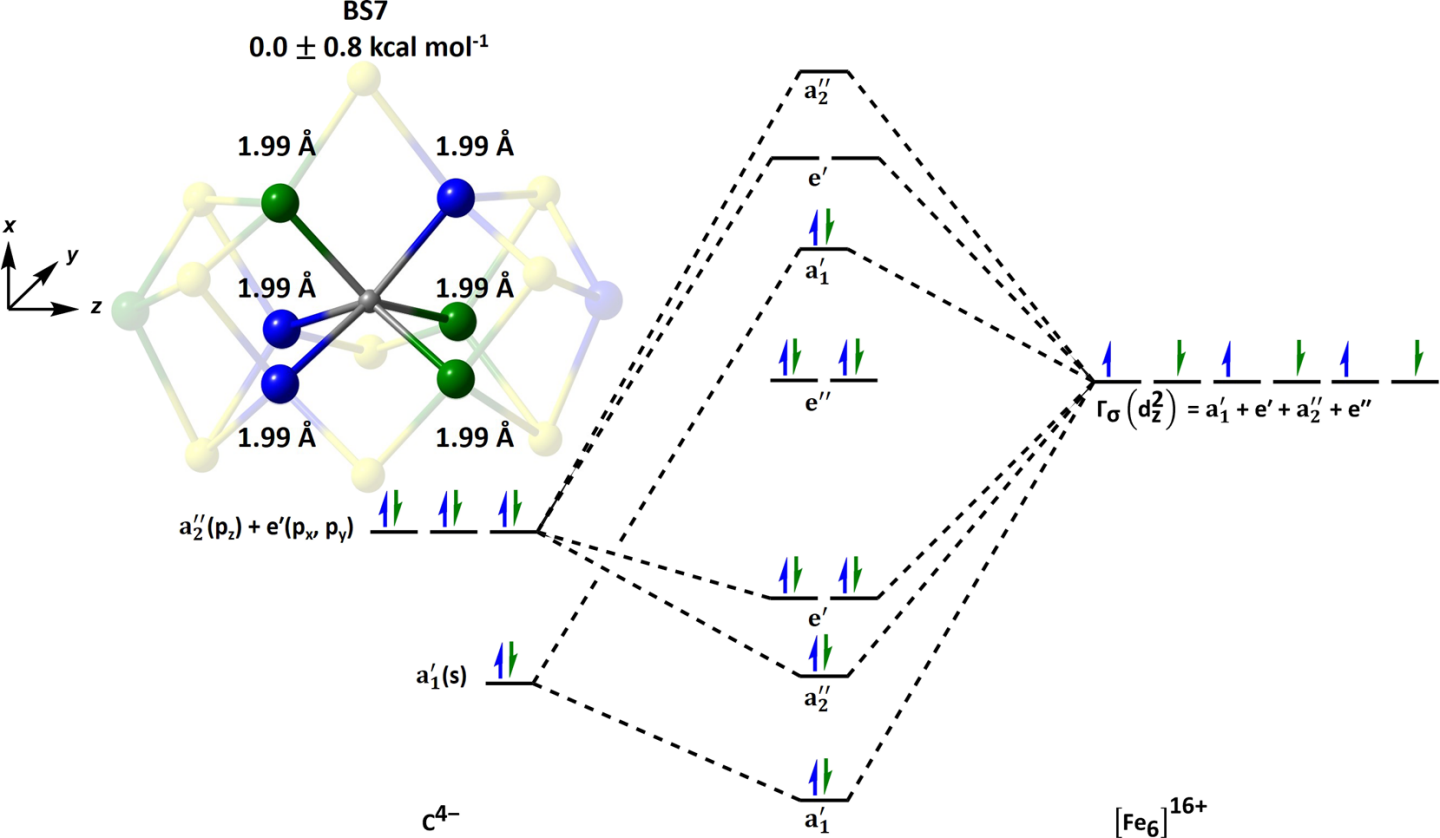
MO diagram for the σ-bonding between the interstitial carbide (left) and Fe d_*z*_^2^ orbitals, with respect to the BS7 solution for FeMoco’s *E*_0_ state. The symmetry labels are with respect to the *D*_3h_ point group.

**Figure 10:**
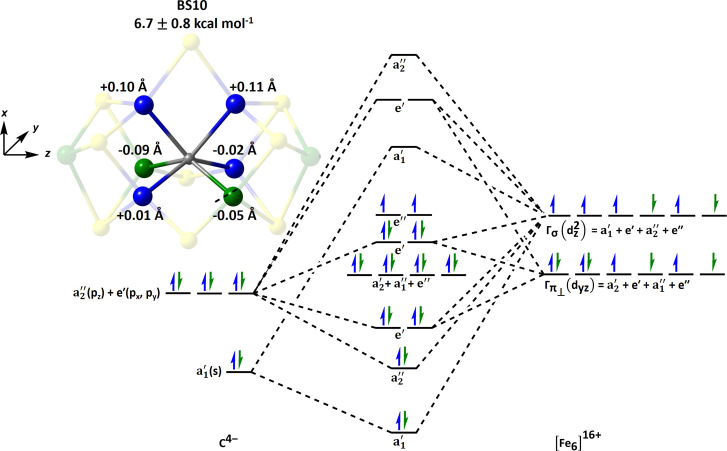
MO diagram for the σ- and π-bonding between the interstitial carbide (left) and Fe SALC’s with σ- and π_⊥_ symmetry (right), with respect to the BS10 solution for FeMoco’s *E*_0_ state. The symmetry labels are with respect to the *D*_3h_ point group.

Comparatively, the BS10 solution has an unequal number of net α- and β-spin centers that can exhibit perpendicular π-bonds with the μ_6_-carbide (Figure 10). The Fe d_*yz*_ orbitals with π_⊥_ symmetry is singly and doubly occupied for respective Fe^3+^ and Fe^2+^ centers. Only the π_⊥_ SALC with e^′^ symmetry can bond to the central carbide, while its four remaining orbitals are nonbonding. The BS10 solution’s MO diagram shows full occupation of bonding orbitals whose e^′^ symmetric orbitals have both σ- and π-components. The e^″^ nonbonding orbitals are singly occupied and localized to the Fe d_*z*_^2^ orbitals that support the negligible spin density at the μ_6_-carbide in the BS10 coupling scheme.

## Conclusion

Our study examines the common ground between the “beating heart” and “heart of steel” models for nitrogenase’s interstitial carbide, which have previously been treated as mutually exclusive. We explore the BS-manifold in FeMoco’s resting state and find that the carbide’s trigonal prismatic geometry and average bond length remain conserved despite dramatic changes to the cluster’s electronic structure. While the average bond length is constant, there are expansions and contractions on the order of 0.1 Å for individual C–Fe bonds. Each BS-solution has negligible net spin density at the central carbide, suggesting the carbide’s experimental hyperfine coupling is not specific to the cofactor’s underlying electronic structure. Using valence bond theory, we detail how the carbide’s hybridization is adaptive to the magnetic coupling of the coordinated Fe-centers. Also illustrated by MO theory, the interstitial carbide can compensate for weakened σ-bonding by strengthened π-bonding that conserves the cofactor’s trigonal prismatic core. We note that our current carbide bonding analysis can be similarly applied to further catalytic intermediates in either Mo-, V-, or Fe-only nitrogenases.

## Supplementary Material





## Data Availability

All other relevant data generated and analyzed during this study are available in the Edmond Open Research Data Repository at 10.17617/3.QM58TQ and in the Supporting Information.

## Notes

QM-region of QM/MM calculations, relative energies of the BS-solutions, spin populations of the BS-solutions localized at the metal centers and carbide, C–Fe bond lengths and trigonal prismatic shape measure of the BS-solutions, population analysis of the carbide localized orbitals of the BS10 solution, and symmetry adapted linear combinations for σ-bonds in a trigonal prismatic environment (PDF)
Coordinates of QM-regions of the BS-solutions (ZIP)

## References

[1] Van
Stappen C., Decamps L., Cutsail G. E., Bjornsson R., Henthorn J. T., Birrell J. A., DeBeer S. (2020). The Spectroscopy of
Nitrogenases. Chem. Rev..

[2] Seefeldt L. C., Yang Z. Y., Lukoyanov D. A., Harris D. F., Dean D. R., Raugei S., Hoffman B. M. (2020). Reduction
of Substrates by Nitrogenases. Chem. Rev..

[3] Einsle O., Rees D. C. (2020). Structural Enzymology
of Nitrogenase Enzymes. Chem. Rev..

[4] Lancaster K. M., Roemelt M., Ettenhuber P., Hu Y., Ribbe M. W., Neese F., Bergmann U., DeBeer S. (2011). X-Ray Emission
Spectroscopy
Evidences a Central Carbon in the Nitrogenase Iron-Molybdenum Cofactor. Science.

[5] Rees J. A., Bjornsson R., Schlesier J., Sippel D., Einsle O., DeBeer S. (2015). The Fe–V Cofactor of Vanadium Nitrogenase Contains
an Interstitial Carbon Atom. Angew. Chem., Int.
Ed..

[6] Decamps L., Rice D. B., DeBeer S. (2022). An Fe_6_C Core in All Nitrogenase
Cofactors. Angew. Chem., Int. Ed..

[7] Mayer S. M., Niehaus W. G., Dean D. R. (2002). Reduction of Short
Chain Alkynes
by a Nitrogenase α-70^Ala^-Substituted MoFe Protein. J. Chem. Soc., Dalton Trans..

[8] Barney B. M., Igarashi R. Y., Dos Santos P. C., Dean D. R., Seefeldt L. C. (2004). Substrate
Interaction at an Iron-Sulfur Face of the FeMo-Cofactor during Nitrogenase
Catalysis. J. Biol. Chem..

[9] Spatzal T., Perez K. A., Einsle O., Howard J. B., Rees D. C. (2014). Ligand
Binding to the FeMo-Cofactor: Structures of CO-Bound and Reactivated
Nitrogenase. Science.

[10] Sippel D., Einsle O. (2017). The Structure of Vanadium Nitrogenase
Reveals an Unusual
Bridging Ligand. Nat. Chem. Biol. 2017 13:9.

[11] Sippel D., Rohde M., Netzer J., Trncik C., Gies J., Grunau K., Djurdjevic I., Decamps L., Andrade S. L. A., Einsle O. (2018). A Bound Reaction Intermediate
Sheds Light on the Mechanism
of Nitrogenase. Science.

[12] Rohde M., Grunau K., Einsle O., Rohde M., Grunau K., Einsle O. (2020). CO Binding to the FeV Cofactor of
CO-Reducing Vanadium
Nitrogenase at Atomic Resolution. Angew. Chem.,
Int. Ed..

[13] Kang W., Lee C. C., Jasniewski A. J., Ribbe M. W., Hu Y. (2020). Structural
Evidence for a Dynamic Metallocofactor during N_2_ Reduction
by Mo-Nitrogenase. Science.

[14] Peters J. W., Einsle O., Dean D. R., DeBeer S., Hoffman B. M., Holland P. L., Seefeldt L. C. (2021). Comment on Structural
Evidence for
a Dynamic Metallocofactor during N_2_ Reduction by Mo-Nitrogenase. Science.

[15] Buscagan T. M., Kaiser J. T., Rees D. C. (2022). Selenocyanate Derived Se-Incorporation
into the Nitrogenase Fe Protein Cluster. eLife.

[16] Trncik C., Detemple F., Einsle O. (2023). Iron-Only Fe-Nitrogenase
Underscores
Common Catalytic Principles in Biological Nitrogen Fixation. Nat. Catal..

[17] Mendel R. R., Einsle O. (2023). On the Shoulders of Giants—Reaching
for Nitrogenase. Molecules.

[18] Lukoyanov D. A., Yang Z. Y., Pérez-González A., Raugei S., Dean D. R., Seefeldt L. C., Hoffman B. M. (2022). ^13^C ENDOR Characterization of the Central Carbon within the Nitrogenase
Catalytic Cofactor Indicates That the CFe_6_Core Is a Stabilizing
“Heart of Steel”. J. Am. Chem.
Soc..

[19] Creutz S. E., Peters J. C. (2014). Catalytic Reduction of N_2_ to NH_3_ by an Fe-N_2_ Complex Featuring a C-Atom Anchor. J. Am. Chem. Soc..

[20] Rittle J., Peters J. C. (2013). Fe-N_2_/CO Complexes That Model a Possible
Role for the Interstitial C Atom of FeMo-Cofactor (FeMoco). Proc. Natl. Acad. Sci. U.S.A..

[21] Van
Stappen C., Benediktsson B., Rana A., Chumakov A., Yoda Y., Bessas D., Decamps L., Bjornsson R., DeBeer S. (2023). Structural Correlations of Nitrogenase Active Sites
Using Nuclear Resonance Vibrational Spectroscopy and QM/MM Calculations. Faraday Discuss..

[22] Scott A. D., Pelmenschikov V., Guo Y., Yan L., Wang H., George S. J., Dapper C. H., Newton W. E., Yoda Y., Tanaka Y., Cramer S. P. (2014). Structural Characterization of CO-Inhibited
Mo-Nitrogenase by Combined Application of Nuclear Resonance Vibrational
Spectroscopy, Extended X-Ray Absorption Fine Structure, and Density
Functional Theory: New Insights into the Effects of CO Binding and
the Role of the Interstitial Atom. J. Am. Chem.
Soc..

[23] Buscagan T.
M., Perez K. A., Maggiolo A. O., Rees D. C., Spatzal T. (2021). Structural
Characterization of Two CO Molecules Bound to the Nitrogenase Active
Site. Angew. Chem., Int. Ed..

[24] Rohde M., Laun K., Zebger I., Stripp S. T., Einsle O. (2021). Two Ligand-Binding
Sites in CO-Reducing V Nitrogenase Reveal a General Mechanistic Principle. Sci. Adv..

[25] Grunenberg J. (2017). The Interstitial
Carbon of the Nitrogenase FeMo Cofactor Is Far Better Stabilized than
Previously Assumed. Angew. Chem., Int. Ed..

[26] Stanghellini P. L., Sailor M. J., Kuznesof P., Whitmire K. H., Hriljac J. A., Kolis J. W., Zheng Y., Shriver D. F. (1987). Vibrational Frequencies
Associated with the Carbide Ligand in Iron Butterfly Clusters. Inorg. Chem..

[27] Pérez-González A., Yang Z. Y., Lukoyanov D. A., Dean D. R., Seefeldt L. C., Hoffman B. M. (2021). Exploring the Role of the Central Carbide of the Nitrogenase
Active-Site FeMo-Cofactor through Targeted ^13^C Labeling
and ENDOR Spectroscopy. J. Am. Chem. Soc..

[28] Le L. N. V., He T., Joyce J. P., Oyala P. H., DeBeer S., Agapie T. (2025). Molybdenum-Iron-Sulfur
Cluster with a Bridging Carbide
Ligand as a Partial FeMoco Model: CO Activation, EPR Studies, and
Bonding Insight. J. Am. Chem. Soc..

[29] Lewis G. N. (1916). The Atom
and the Molecule. J. Am. Chem. Soc..

[30] Pauling L. (1931). The Nature
of the Chemical Bond. Application of Results Obtained from the Quantum
Mechanics and from a Theory of Paramagnetic Susceptibility to the
Structure of Molecules. J. Am. Chem. Soc..

[31] Martin J. C. (1983). “Frozen”
Transition States: Pentavalent Carbon et Al. Science.

[32] Brown, H. C. That Fascinating Nonclassical Ion Problem. In The Nonclassical Ion Problem; Springer, 1977; pp 13–19.

[33] Spatzal T., Aksoyoglu M., Zhang L., Andrade S. L. A., Schleicher E., Weber S., Rees D. C., Einsle O. (2011). Evidence for
Interstitial
Carbon in Nitrogenase FeMo Cofactor. Science.

[34] Benediktsson B., Bjornsson R. (2017). QM/MM Study of the Nitrogenase MoFe
Protein Resting
State: Broken-Symmetry States, Protonation States, and QM Region Convergence
in the FeMoco Active Site. Inorg. Chem..

[35] Bjornsson, R. ASH - A Multiscale Modelling Program; Version 0.9; Springer Nature, 2022.

[36] Eastman P., Swails J., Chodera J. D., McGibbon R. T., Zhao Y., Beauchamp K. A., Wang L.-P., Simmonett A. C., Harrigan M. P., Stern C. D., Wiewiora R. P., Brooks B. R., Pande V. S. (2017). OpenMM 7: Rapid Development of High Performance Algorithms
for Molecular Dynamics. PLoS Comput. Biol..

[37] Neese F. (2018). Software Update:
The ORCA Program System, Version 4.0. Wiley
Interdiscip. Rev. Comput. Mol. Sci..

[38] Neese F., Wennmohs F., Becker U., Riplinger C. (2020). The ORCA Quantum
Chemistry Program Package. J. Chem. Phys..

[39] Sherwood P., de Vries A. H., Guest M. F., Schreckenbach G., Catlow C. R. A., French S. A., Sokol A. A., Bromley S. T., Thiel W., Turner A. J., Billeter S., Terstegen F., Thiel S., Kendrick J., Rogers S. C., Casci J., Watson M., King F., Karlsen E., Sjøvoll M., Fahmi A., Schäfer A., Lennartz C. (2003). QUASI: A General Purpose
Implementation of the QM/MM Approach and Its Application to Problems
in Catalysis. J. Mol. Struct.:THEOCHEM.

[40] Wang L.-P., Song C. (2016). Geometry Optimization
Made Simple with Translation and Rotation Coordinates. J. Chem. Phys..

[41] Billeter S. R., Turner A. J., Thiel W. (2000). Linear Scaling
Geometry Optimisation
and Transition State Search in Hybrid Delocalised Internal Coordinates. Phys. Chem. Chem. Phys..

[42] Furness J.
W., Kaplan A. D., Ning J., Perdew J. P., Sun J. (2020). Accurate and
Numerically Efficient R2SCAN Meta-Generalized Gradient Approximation. J. Phys. Chem. Lett..

[43] Caldeweyher E., Bannwarth C., Grimme S. (2017). Extension of the D3 Dispersion Coefficient
Model. J. Chem. Phys..

[44] Ehlert S., Huniar U., Ning J., Furness J. W., Sun J., Kaplan A. D., Perdew J. P., Brandenburg J. G. (2021). R2SCAN-D4:
Dispersion Corrected Meta-Generalized Gradient Approximation for General
Chemical Applications. J. Chem. Phys..

[45] van
Lenthe E., Baerends E. J., Snijders J. G. (1993). Relativistic Regular
Two-component Hamiltonians. J. Chem. Phys..

[46] van
Wüllen C. (1998). Molecular Density Functional Calculations in the Regular
Relativistic Approximation: Method, Application to Coinage Metal Diatomics,
Hydrides, Fluorides and Chlorides, and Comparison with First-Order
Relativistic Calculations. J. Chem. Phys..

[47] Benediktsson B., Bjornsson R. (2022). Analysis of the Geometric and Electronic Structure
of Spin-Coupled Iron-Sulfur Dimers with Broken-Symmetry DFT: Implications
for FeMoco. J. Chem. Theory Comput..

[48] Weigend F., Ahlrichs R. (2005). Balanced Basis Sets of Split Valence,
Triple Zeta Valence
and Quadruple Zeta Valence Quality for H to Rn: Design and Assessment
of Accuracy. Phys. Chem. Chem. Phys..

[49] Pantazis D. A., Chen X. Y., Landis C. R., Neese F. (2008). All-Electron Scalar
Relativistic Basis Sets for Third-Row Transition Metal Atoms. J. Chem. Theory Comput..

[50] Rolfes J. D., Neese F., Pantazis D. A. (2020). All-Electron Scalar Relativistic
Basis Sets for the Elements Rb–Xe. J.
Comput. Chem..

[51] Neese F. (2003). An Improvement
of the Resolution of the Identity Approximation for the Formation
of the Coulomb Matrix. J. Comput. Chem..

[52] Weigend F., Ahlrichs R. (2005). Balanced Basis Sets
of Split Valence, Triple Zeta Valence
and Quadruple Zeta Valence Quality for H to Rn: Design and Assessment
of Accuracy. Phys. Chem. Chem. Phys..

[53] Hirshfeld F. L. (1977). Bonded-Atom
Fragments for Describing Molecular Charge Densities. Theor. Chim. Acta.

[54] Pipek J., Mezey P. G. (1989). A Fast Intrinsic
Localization Procedure Applicable
for Ab Initio and Semiempirical Linear Combination of Atomic Orbital
Wave Functions. J. Chem. Phys..

[55] Alvarez S., Avnir D., Llunell M., Pinsky M. (2002). Continuous Symmetry
Maps and Shape Classification. The Case of Six-Coordinated Metal Compounds. New J. Chem..

[56] Lovell T., Li J., Liu T., Case D. A., Noodleman L. (2001). FeMo Cofactor
of Nitrogenase: A Density Functional Study of States M^N^, M^OX^, M^R^, and M^I^. J. Am. Chem. Soc..

[57] Bjornsson R., Neese F., DeBeer S. (2017). Revisiting the Mössbauer Isomer
Shifts of the FeMoco Cluster of Nitrogenase and the Cofactor Charge. Inorg. Chem..

[58] Bjornsson R., Lima F. A., Spatzal T., Weyhermüller T., Glatzel P., Bill E., Einsle O., Neese F., DeBeer S. (2014). Identification of a Spin-Coupled Mo­(III) in the Nitrogenase
Iron–Molybdenum Cofactor. Chem. Sci..

[59] Bortoluzzi M., Ciabatti I., Cesari C., Femoni C., Iapalucci M. C., Zacchini S. (2017). Synthesis of the Highly
Reduced [Fe_6_C­(CO)_15_]^4–^ Carbonyl
Carbide Cluster and Its Reactions
with H^+^ and [Au­(PPh_3_)]^+^. Eur. J. Inorg. Chem..

[60] Lukoyanov D., Pelmenschikov V., Maeser N., Laryukhin M., Yang T. C., Noodleman L., Dean D. R., Case D. A., Seefeldt L. C., Hoffman B. M. (2007). Testing If the Interstitial Atom,
X, of the Nitrogenase Molybdenum-Iron Cofactor Is N or C: ENDOR, ESEEM,
and DFT Studies of the S = 3/2 Resting State in Multiple Environments. Inorg. Chem..

[61] Hoeke V., Tociu L., Case D. A., Seefeldt L. C., Raugei S., Hoffman B. M. (2019). High-Resolution ENDOR Spectroscopy Combined with Quantum
Chemical Calculations Reveals the Structure of Nitrogenase Janus Intermediate
E_4_(4H). J. Am. Chem. Soc..

[62] Thorhallsson A. T., Benediktsson B., Bjornsson R. (2019). A Model for Dinitrogen Binding in
the E_4_ State of Nitrogenase. Chem.
Sci..

[63] Cao L., Ryde U. (2020). What Is the Structure
of the E_4_ Intermediate in Nitrogenase?. J. Chem. Theory Comput..

[64] Spiller N., Bjornsson R., DeBeer S., Neese F. (2021). Carbon Monoxide
Binding
to the Iron–Molybdenum Cofactor of Nitrogenase: A Detailed
Quantum Mechanics/Molecular Mechanics Investigation. Inorg. Chem..

[65] Jiang H., Ryde U. (2024). Reaction Mechanism for CO Reduction
by Mo-Nitrogenase Studied by
QM/MM. Inorg. Chem..

[66] Heaton B. T., Iggo J. A., Longoni G., Mulley S. (1995). Solution and Solid-State
Nuclear Magnetic Resonance Studies on Interstitial Atoms within Transition-Metal
Carbonyl Clusters. J. Chem. Soc., Dalton Trans..

[67] Spatzal T., Einsle O., Andrade S. L. A. (2013). Analysis of the
Magnetic Properties
of Nitrogenase FeMo Cofactor by Single-Crystal EPR Spectroscopy. Angew. Chem., Int. Ed..

[68] Hart J. R., Rappé A. K., Gorun S. M., Upton T. H. (1992). Magnetic Interactions
in a Three Center, Four Electron System. J.
Phys. Chem..

[69] Woon D. E., Dunning T. H. (2009). Theory of Hypervalency: Recoupled Pair Bonding in SF_n_ (n = 1–6). J. Phys. Chem. A.

[70] Dunning T. H., Woon D. E., Leiding J., Chen L. (2013). The First Row Anomaly
and Recoupled Pair Bonding in the Halides of the Late P-Block Elements. Acc. Chem. Res..

[71] Vela J., Cirera J., Smith J. M., Lachicotte R. J., Flaschenriem C. J., Alvarez S., Holland P. L. (2007). Quantitative Geometric
Descriptions of the Belt Iron Atoms of the Iron-Molybdenum Cofactor
of Nitrogenase and Synthetic Iron­(II) Model Complexes. Inorg. Chem..

[72] Lancaster K. M., Hu Y., Bergmann U., Ribbe M. W., DeBeer S. (2013). X-Ray Spectroscopic
Observation of an Interstitial Carbide in Nifen-Bound Femoco Precursor. J. Am. Chem. Soc..

