# Supplementary material for: Recasting Nitrogenase’s Carbide Role as a Beating Heart of Steel: A Joint Inorganic and Organic Perspective for μ6Carbide–Iron Bonding
Source: Inorg Chem. 2026 Feb 18;65(9):5028–36. doi: 10.1021/acs.inorgchem.5c05356 (PMC12977034; doi:10.1021/acs.inorgchem.5c05356)
Supplement: Supplementary file 1 [file ic5c05356_si_001.pdf]

Supporting Information

Recasting Nitrogenase's Carbide Role as a Beating Heart of Steel: A Joint Inorganic and Organic Perspective for μ_6Carbide-Iron Bonding

Justin P. Joyce* and Serena DeBeer*

Justin P. Joyce – *Department of Inorganic Spectroscopy, Max Planck Institute for Chemical Energy Conversion, Stiftstr. 34-36, Mülheim an der Ruhr D-45470, Germany*

Serena DeBeer – *Department of Inorganic Spectroscopy, Max Planck Institute for Chemical Energy Conversion, Stiftstr. 34-36, Mülheim an der Ruhr D-45470, Germany*

*Email: justin.joyce@cec.mpg.de

*Email: serena.debeer@cec.mpg.de

Table of Contents:

I. Computational Setup (S1)
II. Broken-Symmetry Analysis (S2)
III. Symmetry Adapted Linear Combinations (S4)

SI. Computation Setup

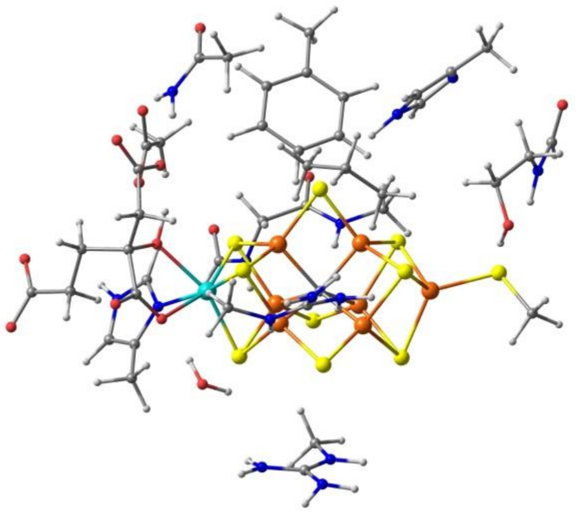

Figure S1. The QM-region of FeMoco, illustrated in its BS-235M optimized E_0 state. The residue identities are Val70, Arg96, Gln191, His195, Cys275, Ser278, Gly356, Gly357, Arg359, Glu380, Phe381, His442 and HOH519.

SII. Broken-Symmetry Analysis.

Table S1. The relative energy for the QM(r²SCAN)/MM optimized geometries for the BS-solutions of FeMoco's E_0 state. The BS-solutions are labeled by their Noodleman class, while our notation is provided in parentheses.

BS-Solution	E (kcal mol^{-1})
BS1 (567)	30.93
BS2 (234)	11.47
BS3 (123)	27.17
BS3 (124)	23.73
BS3 (134)	25.70
BS4 (257)	15.32
BS4 (356)	13.94
BS4 (467)	34.34
BS5 (256)	18.68
BS5 (357)	18.07
BS5 (367)	19.40
BS5 (456)	21.43
BS5 (457)	41.14
BS5 (267)	20.48
BS6 (156)	7.68
BS6 (157)	8.19
BS6 (167)	7.83
BS7 (235)	0.00
BS7 (247)	1.49
BS7 (346)	0.13
BS8 (245)	8.44
BS8 (345)	6.83
BS8 (236)	7.01
BS8 (246)	9.08
BS8 (237)	6.65
BS8 (347)	6.94
BS9 (126)	12.64
BS9 (137)	13.81
BS9 (145)	11.90
BS10 (127)	6.48
BS10 (136)	8.25
BS10 (135)	8.28
BS10 (147)	6.65
BS10 (125)	6.83
BS10 (146)	7.08

Table S2. The Hirshfeld spin populations for the metal centers and interstitial carbide for the QM(r²SCAN)/MM optimized geometries for the BS-solutions of FeMoco's E_0 state. The BS-solutions are labeled by their Noodleman class, while our notation is provided in parentheses.

BS-Solution	Mo	Fe1	Fe2	Fe3	Fe4	Fe5	Fe6	Fe7	μ_6C
BS1 (567)	0.56α	2.62β	3.25α	3.21α	3.15α	2.13β	1.22β	1.86β	0.14α
BS2 (234)	0.89β	3.11α	2.53β	2.66β	2.61β	2.69α	2.73α	3.68α	0.05α
BS3 (123)	0.90β	2.83β	1.92β	2.96β	2.96α	2.88α	2.78α	2.78α	0.02β
BS3 (124)	0.91β	2.69β	2.06β	2.91α	2.94β	2.86α	2.78α	2.78α	0.01β

BS3 (134)	0.87β	2.80β	2.89α	1.97β	2.96β	2.86α	2.75α	2.80α	0.01β
BS4 (257)	0.20α	2.80α	2.88β	2.11α	2.99α	2.65β	2.66α	2.54β	0.01β
BS4 (356)	0.29α	2.44α	2.62α	2.91β	2.87α	2.66β	2.53β	2.64α	0.01β
BS4 (467)	0.29β	0.50α	3.09α	3.10α	2.55β	2.84α	2.42β	2.45β	0.08α
BS5 (256)	0.27α	3.06α	2.89β	2.25α	3.02α	2.78β	2.70β	2.53α	0.01β
BS5 (357)	0.19α	3.09α	2.14α	2.92β	3.02α	2.75β	2.55α	2.69β	0.01β
BS5 (367)	0.31α	2.42α	2.81α	2.91β	2.98α	2.65α	2.72β	2.68β	0.01β
BS5 (456)	0.31α	3.12α	3.01α	1.94α	2.91β	2.75β	2.64β	2.59α	0.01β
BS5 (457)	0.04α	0.55α	2.95α	2.95α	2.67β	1.80β	2.69α	2.25β	0.06α
BS5 (267)	0.27α	2.92α	2.89β	2.31α	3.07α	2.65α	2.72β	2.72β	0.01β
BS6 (156)	0.30α	3.14β	2.71α	2.93α	2.79α	2.62β	2.56β	2.60α	0.04β
BS6 (157)	0.26α	3.15β	2.85α	2.80α	2.79α	2.62β	2.58α	2.58β	0.04β
BS6 (167)	0.32α	3.15β	2.70α	2.78α	2.92α	2.66α	2.56β	2.57β	0.05β
BS7 (235)	0.32β	3.16α	2.78β	2.83β	3.01α	2.76β	2.56α	2.57α	0.00
BS7 (247)	0.25β	3.13α	2.77β	2.99α	2.83β	2.64α	2.53α	2.72β	0.00
BS7 (346)	0.17β	3.14α	2.96α	2.84β	2.86β	2.64α	2.70β	2.55α	0.00
BS8 (245)	0.30β	3.11α	2.91β	3.00α	2.97β	2.68β	2.67α	2.71α	0.00
BS8 (345)	0.30β	3.12α	2.95α	2.96β	3.00β	2.65β	2.72α	2.69α	0.00
BS8 (236)	0.19β	3.12α	2.96β	2.97β	3.02α	2.77α	2.65β	2.67α	0.00
BS8 (246)	0.19β	3.10α	2.94β	2.98α	2.96β	2.73α	2.62β	2.70α	0.00
BS8 (237)	0.28β	3.14α	2.96β	3.00β	3.03α	2.78α	2.67α	2.64β	0.00
BS8 (347)	0.26β	3.12α	2.93α	2.99β	2.98β	2.75α	2.70α	2.58β	0.00
BS9 (126)	0.23β	3.17β	3.03β	3.07α	3.08α	2.88α	2.56β	2.82α	0.03β
BS9 (137)	0.27β	3.09β	2.94α	3.00β	2.99α	2.84α	2.78α	2.47β	0.00
BS9 (145)	0.31β	3.11β	2.96α	3.01α	3.00β	2.54β	2.81α	2.79α	0.01β
BS10 (127)	0.33β	3.12β	2.91β	2.92α	3.13α	2.95α	2.98α	2.53β	0.01α
BS10 (136)	0.27β	3.11β	2.86α	2.95β	3.11α	2.94α	2.49β	2.70α	0.02α
BS10 (135)	0.38β	3.12β	3.07α	2.97β	2.92α	2.52β	2.90α	2.71α	0.01α
BS10 (147)	0.33β	3.14β	3.09α	2.93α	2.98β	2.75α	2.87α	2.50β	0.01α
BS10 (125)	0.38β	3.12β	2.92β	3.12α	2.94α	2.56β	2.70α	2.89α	0.01α
BS10 (146)	0.25β	3.14β	2.90α	3.12α	2.97β	2.75α	2.51β	2.86α	0.01α

Table S3. The carbide-iron bond length and trigonal prismatic continuous shape measure for the carbide core of the QM(r^{2}SCAN)/MM optimized geometries for the BS-solutions of FeMoco's E_0 state. The BS-solutions are labeled by their Noodleman class, while our notation is provided in parentheses.

BS-Solution	C-Fe2 (Å)	C-Fe3 (Å)	C-Fe4 (Å)	C-Fe5 (Å)	C-Fe6 (Å)	C-Fe7 (Å)	CShM (TRP-6)
BS1 (567)	2.14	2.13	2.05	1.87	1.87	1.87	0.23
BS2 (234)	1.94	1.95	1.95	1.95	2.13	1.98	0.18
BS3 (123)	1.88	1.96	2.02	2.13	2.03	1.96	0.18
BS3 (124)	1.91	1.99	1.96	1.96	2.06	2.12	0.23
BS3 (134)	2.00	1.90	1.96	1.98	2.13	2.02	0.23
BS4 (257)	1.98	1.98	2.00	1.99	1.98	1.96	0.05
BS4 (356)	2.00	2.00	2.01	1.98	1.98	1.98	0.04
BS4 (467)	2.03	2.02	1.99	2.01	1.98	1.97	0.13
BS5 (256)	1.98	1.99	1.96	1.96	2.10	1.94	0.14
BS5 (357)	1.95	1.99	1.97	1.95	1.97	2.09	0.09
BS5 (367)	1.98	1.98	2.08	1.93	1.96	2.04	0.13
BS5 (456)	1.96	1.91	1.99	2.09	1.98	1.98	0.10
BS5 (457)	2.13	2.01	1.94	1.87	2.01	1.93	0.15
BS5 (267)	1.97	1.95	2.06	1.93	2.07	1.95	0.18
BS6 (156)	1.98	2.11	1.99	1.94	1.96	1.94	0.10

BS6 (157)	2.09	1.99	1.99	1.94	1.95	1.95	0.09
BS6 (167)	1.97	1.98	2.12	1.95	1.96	1.94	0.11
BS7 (235)	1.98	1.99	1.99	1.97	2.01	2.00	0.04
BS7 (247)	1.98	1.99	1.99	2.00	2.00	1.97	0.03
BS7 (346)	1.99	1.99	1.99	1.99	1.98	1.99	0.01
BS8 (245)	1.96	1.97	2.07	1.95	1.97	2.05	0.11
BS8 (345)	1.96	1.97	2.08	1.93	2.07	1.96	0.13
BS8 (236)	2.06	1.96	1.97	2.07	1.96	1.96	0.11
BS8 (246)	2.06	1.97	1.97	1.96	1.95	2.06	0.09
BS8 (237)	1.96	2.07	1.97	2.07	1.97	1.95	0.11
BS8 (347)	1.95	2.08	1.97	1.95	2.08	1.93	0.13
BS9 (126)	1.94	2.05	2.05	2.03	1.90	2.02	0.06
BS9 (137)	2.03	1.96	2.04	2.03	2.05	1.89	0.09
BS9 (145)	2.03	2.05	1.95	1.89	2.05	2.03	0.11
BS10 (127)	1.94	1.98	2.10	2.08	2.01	1.90	0.10
BS10 (136)	1.97	1.94	2.10	2.09	1.91	1.99	0.18
BS10 (135)	2.09	1.95	1.98	1.89	2.11	2.00	0.15
BS10 (147)	2.09	1.97	1.95	1.99	2.11	1.90	0.15
BS10 (125)	1.94	2.10	1.98	1.90	2.01	2.08	0.11
BS10 (146)	1.97	2.11	1.95	2.00	1.91	2.09	0.12

Table S4. The Loewdin Population analysis of the $\mu_6$carbide PM-localized orbitals for the BS-127 optimized solution, which is representative of the BS10 class. The orbitals are labeled and normalized with respect to the CFe_6 fragment.

BS-Solution	μ_6C (s)	μ_6C (p)	Fe2 (d)	Fe3 (d)	Fe4 (d)	Fe5 (d)	Fe6 (d)	Fe7 (d)
**sp^n (α)**	46.2	15.7	9.8	4.0	4.2	4.0	4.0	12.2
	5.7	39.7	9.7	2.2	1.6	1.0	2.0	38.2
	5.8	41.0	30.3	2.2	1.0	1.6	2.1	16.1
**sp^n (β)**	22.2	31.1	2.9	5.7	15.6	15.1	4.4	3.0
	13.3	32.0	2.5	39.9	1.8	3.7	4.7	2.2
	13.5	33.9	1.9	6.0	5.7	2.2	33.9	2.8
**p_z (α)**	0.3	52.1	18.1	1.4	1.5	1.8	1.4	23.4
**p_z (β)**	0.1	54.0	1.9	12.8	8.2	9.3	11.9	1.8

<u>SIII. Symmetry Adapted Linear Combinations.</u>

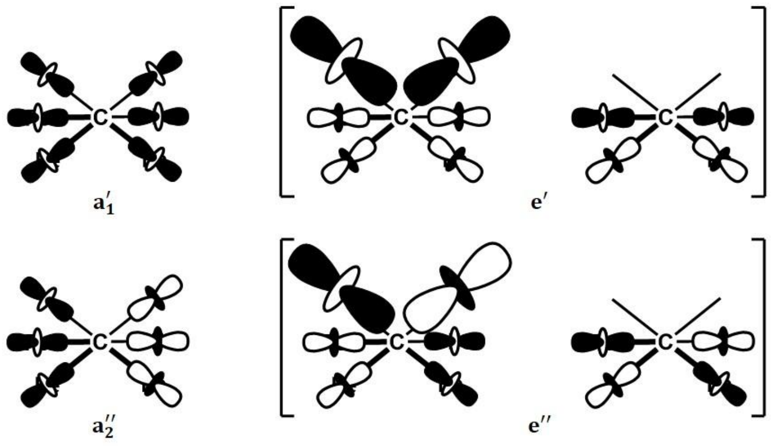

Figure S2. The symmetry adapted linear combinations (SALCs) of the Fe-centers d_{z^2} orbitals that σ-bond with the carbide core.
